# Editorial: Mechanisms guarding the genome

**DOI:** 10.3389/fcell.2022.974545

**Published:** 2022-08-15

**Authors:** James A. L. Brown, E Bourke, W. W Hancock, D. J Richard

**Affiliations:** ^1^ Department of Biological Sciences, University of Limerick, Limerick, Ireland; ^2^ Limerick Digital Cancer Research Centre, HRI, ULCaN, University of Limerick, Limerick, Ireland; ^3^ Lambe Institute for Translational Research, Discipline of Pathology, Centre for Chromosome Biology, National University of Ireland, Galway, Ireland; ^4^ Department of Pathology and Laboratory Medicine, University of Pennsylvania, Philadelphia, PA, United States.; ^5^ Cancer and Ageing Research Program, School of Biomedical Sciences, Institute of Health and Biomedical Innovation, Translational Research Institute, Queensland University of Technology, Brisbane, QLD, Australia

**Keywords:** stability, chromatin bridges, hypoxia, TIP60, ATM, DDR1, Trex1, RAD51AP1

The genomic integrity of our cells is critical to their normal function, and is protected though the activity of many diverse and essential signalling pathways, with dysregulation of these pathways leading to increasing levels of genomic instability (Hanahan and Weinberg, 2011; Kass et al., 2016; Hanahan, 2022). Genomic instability is a recognized hallmark of cancer (Negrini et al., 2010; Hanahan, 2022), and is known to drive tumourigenesis though its impact on mutations, chromatin organisation, and the dysregulation of gene regulation, facilitating tumour development (Aguilera and Gómez-González, 2008; Burrell et al., 2013). Key molecular mechanisms and processes regulating genome stability include the DNA damage response (DDR), epigenetic reprogramming, and organelle abnormalities (e.g. centrosome amplification) (Ciccia and Elledge, 2010; Bettencourt-Dias et al., 2011; Suvà et al., 2013). Improved understanding of these intrinsic cancer-specific mechanisms informs the development, and application of, the next generation targeted therapeutics (J.A.L Brown J. A. L. et al., 2016; Kraus, 2018; Matchett et al., 2017; Prakash et al., 2018).

The review *Tumor Hypoxia Drives Genomic Instability* by Tang et al. explores the mechanisms associated altered and dysregulated in the hypoxic tumour environment. The paper focuses on how tumour hypoxia induces genome instability, though activation and modulation of DNA damage responses (including double strand break repair, mismatch Repair and Base excision repair). The paper also explores the effects of hypoxia on therapeutic treatment responses, and thier manipulation to maximize therapeutic effects of current and future treatments.

Chromatin bridges can form from alterations in DNA metabolism (including chromosome mis-segregation) are resolved before cells division. *The Last Chance Saloon* by Hong et al. reviews the consequences of chromatin bridges on chromosome segregation, cellular replication and genomic instability. They detail the processes leading to chromatin bridge formation, the cellular responses when chromatin bridges are detected (abscission checkpoint activation), and the how chromatin bridges are processed (by the TREX1 exonuclease and LEM-3/ANKLE1 endonucleases). They highlight the role and elements of the NoCut checkpoint involved in protecting genome stability though the management of chromatin bridges, and how father investigation of these nucleases may be relevant of many solid tumour types.

The review by Chen et al. *Recent Advances in the Role of Discoidin Domain Receptor Tyrosine Kinase 1 and Discoidin Domain Receptor Tyrosine Kinase 2 in Breast and Ovarian Cancer* concentrates on discussing the role of the transmembrane Discoidin domain receptor tyrosine kinases (DDRs). The review discusses the activation of the kinase activity DDR1 and DDR2 to regulate MAPK signaling, Notch signaling pathways and alter the tumour microenvironment influencing cell invasion and metastasis. The authors highlight the role of DDR1 and DDR2 in breast and ovarian tumour development and progression, and how their dysregulation can alter treatment responses.

Homologues recombination (HR) is a high-fidelity mechanism for protecting genome integrity from double strand breaks (Chapman et al., 2012; Ranjha et al., 2018). HR is frequently altered in many cancers, making it high priority target for the development of new therapeutics (Gent et al., 2001; Chernikova et al., 2012; Sun et al., 2020). The research article *RAD51AP1 and RAD54L can underpin two distinct RAD51-dependent routes of DNA damage repair* via *homologous recombination* by Selemenakis et al. identifies and explores differential roles for RAD51AP1 and RAD54L in homologues recombination, though RAD51-dependent signalling mechanisms. They reveal the existence of the RAD51AP1- and RAD54L-dependent HR sub-pathways, and show that RAD51AP1 can compensate for RAD54L loss. Importantly, the demonstrate that cell deficient in RAD51AP1 and RAD54L are sensitized to the PARP inhibitor Olaparib.

Ataxia Telangiectasia Mutated (ATM) is a key regulator of the DNA double strand break response (DDR), protecting genome integrity against DNA double strand breaks (Clouaire et al., 2017; Price and D’Andrea, 2013). Importantly, the activation of ATM’s DDR-dependent activity is primarily regulated by acetylation from the lysine acetyltransferase Tip60 (Sun et al., 2005; Bakkenist and Kastan, 2015; James A.L.; Brown JA. L. et al., 2016). Likhatcheva et al. used a combination of a Tip60-targeted inhibitor (TH 1834) (Gao et al., 2014) and siRNA explore the ATM-Tip60 triggered signaling dependencies under hypoxic conditions (0.1% oxygen), in *A Novel Mechanism of Ataxia Telangiectasia Mutated Mediated Regulation of Chromatin Remodeling in Hypoxic Conditions*. They found ATM activation (pS 1981) under hypoxic stress does require Tip60 activity, in a H3K9me3 positive heterochromatic state. Under these hypoxic conditions, activated ATM regulated H3K9me3 levels through the downregulation of MDM2, which protects Suv39H1 levels (facilitating Suv39H1-dependent H3K9me3). This work reveals the importance of understanding changes to genomic integrity signaling cascades in a hypoxic environment (which better reflects the intra-tumour environment), which will inform new anti-cancer treatment strategies and options.

The research article *The E3 Ubiquitin Ligase NEDD4L Targets OGG1 for Ubiquitylation and Modulates the Cellular DNA Damage Response* by Hughes and Parsons investigated the role of OGG1 (8-Oxoguanine DNA glycosylase) in protecting genome stability through the base excision repair (BER) pathway. The BER pathway protects the genome from reactive oxygen is 8-oxoguanine (8-oxoG) induced lesions, which can impair DNA replication and genomic integrity (Tubbs and Nussenzweig, 2017). Here the mechanisms regulating OGG1 in response to oxidative stress were examined. They found that NEDD4-like (NEDD4L) was a E3 ubiquitin-protein ligase, and bound to OGG1. *In vitro* NEDD4L ubiquitylates lysine 341 of OGG1, inhibiting its DNA glycosylase/lyase activity. Ionizing radiation (IR) induced oxidative stress, which enriched OGG1 levels, decreasing irradiated cells survival while conversely increasing their DNA repair capacity. This suggests that OGG1 mediates the formation intermediate DNA lesions which are reduce cellular survival. This work reveals how OGG1 protein mediates BER, maintaining genome stability and influencing cell survival.

This collection of research papers and reviews highlight the importance of how understanding the intrinsic cellular environment impacts on genome integrity, by mediating the choice and function of DNA repair pathways. This is illustrated by the research papers exploring the effects of hypoxia on genome integrity signalling. We anticipate this collection will be of interest to both researchers and clinician scientists, and highlights new avenues and targets for therapeutic development as anti-cancer treatments.

## References

[B1] Aguilera A. Gómez-González B. (2008). Genome instability: A mechanistic view of its causes and consequences.. Nat. Rev. Genet. 9, 204–217. 10.1038/nrg2268 18227811

[B2] Bakkenist C. J. Kastan M. B. (2015). Chromatin perturbations during the DNA damage response in higher eukaryotes. DNA repair 36, 8–12. 10.1016/j.dnarep.2015.09.002 26391293 PMC4727245

[B3] Bettencourt-Dias M. Hildebrandt F. Pellman D. Woods G. Godinho S. A. (2011). Centrosomes and cilia in human disease.. Trends Genet. 27, 307–315. 10.1016/j.tig.2011.05.004 21680046 PMC3144269

[B4] Brown J. A. L. Bourke E. Eriksson L. A. Kerin M. J. (2016a). Targeting cancer using KAT inhibitors to mimic lethal knockouts.. Biochem. Soc. Trans. 44, 979–986. 10.1042/BST20160081 27528742 PMC4984449

[B5] Brown J. A. L. Chonghaile T. N. Matchett K. B. Lynam-Lennon N. Kiely P. A. (2016b). Cancer Res.. 10.1158/0008-5472.CAN-16-0860 27803103

[B6] Burrell R. A. McGranahan N. Bartek J. Swanton C. (2013). The causes and consequences of genetic heterogeneity in cancer evolution.. Nature 501, 338–345. 10.1038/nature12625 24048066

[B7] Chapman J. R. Taylor M. R. G. Boulton S. J. (2012). Playing the end game: DNA double-strand break repair pathway choice.. Mol. Cell 47, 497–510. 10.1016/j.molcel.2012.07.029 22920291

[B8] Chernikova S. B. Game J. C. Brown J. M. (2012). Inhibiting homologous recombination for cancer therapy.. Cancer Biol. Ther. 13, 61–68. 10.4161/cbt.13.2.18872 22336907 PMC3336066

[B9] Ciccia A. Elledge S. J. (2010). The DNA damage response: Making it safe to play with knives.. Mol. Cell 40, 179–204. 10.1016/j.molcel.2010.09.019 20965415 PMC2988877

[B10] Clouaire T. Marnef A. Legube G. (2017). Taming tricky DSBs: ATM on duty.. Dna Repair 56, 84–91. 10.1016/j.dnarep.2017.06.010 28624372

[B11] Gao C. Bourke E. Scobie M. Famme M. A. Koolmeister T. Helleday T. (2014). Rational design and validation of a Tip60 histone acetyltransferase inhibitor. Sci. Rep. 4, 5372. 10.1038/srep05372 24947938 PMC4064327

[B12] Gent D. C. van Hoeijmakers J. H. Kanaar R. (2001). Chromosomal stability and the DNA double-stranded break connection. Nat. Rev. Genet. 2, 196–206. 10.1038/35056049 11256071

[B13] Hanahan D. (2022). Hallmarks of cancer: New dimensions.. Cancer Discov. 12, 31–46. 10.1158/2159-8290.CD-21-1059 35022204

[B14] Hanahan D. Weinberg R. A. (2011). Hallmarks of cancer: The next generation.. Cell 144, 646–674. 10.1016/j.cell.2011.02.013 21376230

[B15] Kass E. M. Moynahan M. E. Jasin M. (2016). When genome maintenance goes badly awry.. Mol. Cell 62, 777–787. 10.1016/j.molcel.2016.05.021 27259208 PMC4966655

[B16] Kraus V. B. (2018). Biomarkers as drug development tools: Discovery, validation, qualification and use.. Nat. Rev. Rheumatol. 14, 354–362. 10.1038/s41584-018-0005-9 29760435

[B17] Matchett K. B. Lynam-Lennon N. Watson R. W. Brown J. A. L. (2017). Cancers, 1–8. 10.3390/cancers9110146 PMC5704164 29068364

[B18] Negrini S. Gorgoulis V. G. Halazonetis T. D. (2010). Genomic instability--an evolving hallmark of cancer.. Nat. Rev. Mol. Cell Biol. 11, 220–228. 10.1038/nrm2858 20177397

[B19] Prakash A. Garcia-Moreno J. F. Brown J. A. L. Bourke E. (2018). Clinically applicable inhibitors impacting genome stability. Molecules 23, E1166. 10.3390/molecules23051166 29757235 PMC6100577

[B20] Price B. D. D’Andrea A. D. (2013). Chromatin remodeling at DNA double-strand breaks.. Cell 152, 1344–1354. 10.1016/j.cell.2013.02.011 23498941 PMC3670600

[B21] Ranjha L. Howard S. M. Cejka P. (2018). Main steps in DNA double-strand break repair: An introduction to homologous recombination and related processes.. Chromosoma 127, 187–214. 10.1007/s00412-017-0658-1 29327130

[B22] Sun Y. Jiang X. Chen S. Fernandes N. Price B. D. (2005). A role for the Tip60 histone acetyltransferase in the acetylation and activation of ATM.. Proc. Natl. Acad. Sci. U. S. A. 102, 13182–13187. 10.1073/pnas.0504211102 16141325 PMC1197271

[B23] Sun Y. McCorvie T. J. Yates L. A. Zhang X. (2020). Structural basis of homologous recombination.. Cell. Mol. Life Sci. 77, 3–18. 10.1007/s00018-019-03365-1 31748913 PMC6957567

[B24] Suvà M. L. Riggi N. Bernstein B. E. (2013). Epigenetic reprogramming in cancer.. Science 339, 1567–1570. 10.1126/science.1230184 23539597 PMC3821556

[B25] Tubbs A. Nussenzweig A. (2017). Endogenous DNA damage as a source of genomic instability in cancer.. Cell 168, 644–656. 10.1016/j.cell.2017.01.002 28187286 PMC6591730

